# Microbial Transglutaminase Improves *ex vivo* Adhesion of Gelatin Methacryloyl Hydrogels to Human Cartilage

**DOI:** 10.3389/fmedt.2021.773673

**Published:** 2021-11-18

**Authors:** Anna Trengove, Serena Duchi, Carmine Onofrillo, Cathal D. O'Connell, Claudia Di Bella, Andrea J. O'Connor

**Affiliations:** ^1^Department of Biomedical Engineering, University of Melbourne, Melbourne, VIC, Australia; ^2^Aikenhead Centre for Medical Discovery (ACMD), St Vincent's Hospital Melbourne, Melbourne, VIC, Australia; ^3^Department of Surgery, The University of Melbourne, Melbourne, VIC, Australia; ^4^Discipline of Electrical and Biomedical Engineering, School of Engineering, Royal Melbourne Institute of Technology (RMIT) University, Melbourne, VIC, Australia; ^5^Department of Orthopaedics, St Vincent's Hospital Melbourne, Melbourne, VIC, Australia

**Keywords:** bioadhesive, cartilage repair, transglutaminase, GelMA, integration, tissue engineering, hydrogel, graft

## Abstract

Current surgical techniques to treat articular cartilage defects fail to produce a satisfactory long-term repair of the tissue. Regenerative approaches show promise in their ability to generate hyaline cartilage using biomaterials in combination with stem cells. However, the difficulty of seamlessly integrating the newly generated cartilage with the surrounding tissue remains a likely cause of long-term failure. To begin to address this integration issue, our strategy exploits a biological enzyme (microbial transglutaminase) to effect bioadhesion of a gelatin methacryloyl implant to host tissue. Mechanical characterization of the bioadhesive material shows that enzymatic crosslinking is compatible with photocrosslinking, allowing for a dual-crosslinked system with improved mechanical properties, and a slower degradation rate. Biocompatibility is illustrated with a 3D study of the metabolic activity of encapsulated human adipose derived stem cells. Furthermore, enzymatic crosslinking induced by transglutaminase is not prevented by the presence of cells, as measured by the bulk modulus of the material. Adhesion to human cartilage is demonstrated *ex vivo* with a significant increase in adhesive strength (5.82 ± 1.4 kPa as compared to 2.87 ± 0.9 kPa, *p* < 0.01) due to the addition of transglutaminase. For the first time, we have characterized a bioadhesive material composed of microbial transglutaminase and GelMA that can encapsulate cells, be photo crosslinked, and bond to host cartilage, taking a step toward the integration of regenerative implants.

## Introduction

Effective long-term treatment of damaged articular cartilage is a significant unmet medical challenge (1–3). Cartilage lesions have been observed at rates of ~60% in patients undergoing knee arthroscopy, including in a review of 31,516 knee arthroscopy procedures (noting over 660,000 knee arthroscopies were performed annually in the USA in the mid-1990s, which rose to over 980,000 a year in 2006) (4–6). Focal cartilage defects caused by traumatic knee injuries can be a precursor to post-traumatic osteoarthritis, with the likelihood of knee replacement more than doubling in the 10 to 15 years following a knee-related sporting injury (7, 8). Whilst current surgical treatments to repair cartilage defects can provide relief in the short term, they often result in mechanically weak and inferior fibrocartilage, and see deterioration in the long term (9).

Tissue engineering approaches to repair damaged articular cartilage show promise as a treatment method (9–11). Surgical techniques utilizing cell-free scaffolds, often combined with microfracture, have seen clinical results improve in the short and mid-term, and scaffolds utilizing autologous chondrocytes are on the market in some European and Asian countries (9, 12). Information on the long-term efficacy of cell-free approaches is lacking in the literature, and this remains to be seen for cell-seeded scaffold approaches, though a 5-year follow-up of treatment with autologous chondrocytes cultured on porcine collagen membrane showed promising results relative to microfracture (10, 12, 13).

A major difficulty in achieving long-term repair of cartilage is seamlessly integrating the implanted or newly generated tissue with the surrounding native cartilage—this is essential to recapitulate a smooth and mechanically strong articular surface (14–16). Integration is difficult to achieve in the first instance, as cartilage is by its nature dense and anti-adhesive, and well-understood to have limited ability for self-repair (15, 17). Gaps between neocartilage and the native tissue are worsened by the loading conditions of the knee joint (16, 18–21). Following injury, the joint is also in a persistent inflammatory state, with elevated levels of pro-inflammatory cytokines that mediate the break down of cartilage matrix (22). These factors combined contribute to further degradation of the tissue and likely failure of the repair in the long term, and have been reviewed in more detail (14–17).

One strategy that may overcome the challenge of integration is the use of an adhesive to secure a scaffold or graft to the surrounding host cartilage at the time of implantation, and stabilize the graft during joint movement. Sealing off the defect can also protect the exposed cartilage matrix and nearby chondrocytes from inflammatory factors present in synovial fluid that may lead to further degradation (23). Fibrin based surgical sealants (e.g., Tisseel^®^) have been used to fill chondral and osteochondral defects, however studies have shown poor results, even impairing the natural healing of the defect observed in a rabbit model (24, 25). A chondroitin sulfate adhesive has been used with a polyethylene glycol/hyaluronic acid hydrogel (ChonDux^TM^) in cartilage defects following microfracture (26). A Phase II clinical study of this material showed good filling of the defect at 2-year follow-up; however, evidence of delamination was observed in ~1 quarter of patients.

Rather than an adhesive that is applied prior to implantation or a sealant that is applied after, both of which add complexity and potential variability, there is a need for a one-pot biocompatible and biodegradable material that can act as a scaffold to both deliver stem cells to focal cartilage defects, and to bond itself to the surface of the cartilage in the short term, right after implantation. This cell-laden material should adhere to the tissue without impeding the generation of new matrix and linkages with the surrounding cartilage which in turn is required for the robust integration of the engineered tissue in the long term.

Gelatin methacryloyl (GelMA) is a naturally-derived, photocrosslinkable hydrogel with tunable mechanical properties and good biocompatibility (27). A number of cartilage tissue engineering strategies have employed GelMA as a scaffold for chondrocytes or mesenchymal stromal cells (MSCs) (27). An *in situ* bioprinting approach used a GelMA-hyaluronic acid blend to deliver allogeneic adipose-derived MSCs to focal cartilage defects in a sheep model (28). Evidence of regenerated cartilage was seen after 8 weeks, with a larger quantity observed in the *in situ* bioprinted group compared to microfracture or bench-fabricated and implanted controls, however integration of the neocartilage remained an issue (28). GelMA synthesized with a secondary functionalization for binding to cartilage has also been developed, and was seen to support formation of neocartilage *in vitro* by embedded articular chondroprogenitor cells (29).

Transglutaminases are enzymes that catalyze covalent bonds between the γ-carbonyl and ε-amino groups of glutamine and lysine residues, respectively, found in proteins (30). Various transglutaminases are found in tissues and body fluids, and are involved in processes like blood clotting and wound healing. Microbial transglutaminase, produced by microorganisms, has a number of established applications in food processing and is permitted as a food additive in many countries (31). The United States Food and Drug Administration (FDA) also classifies the enzyme as “Generally Recognized as Safe” (31). Compared to tissue derived/mammalian transglutaminases, microbial transglutaminase does not require calcium ions for activation, is stable over a broad range of temperatures, and is relatively low-cost (31).

**Scheme 1:**
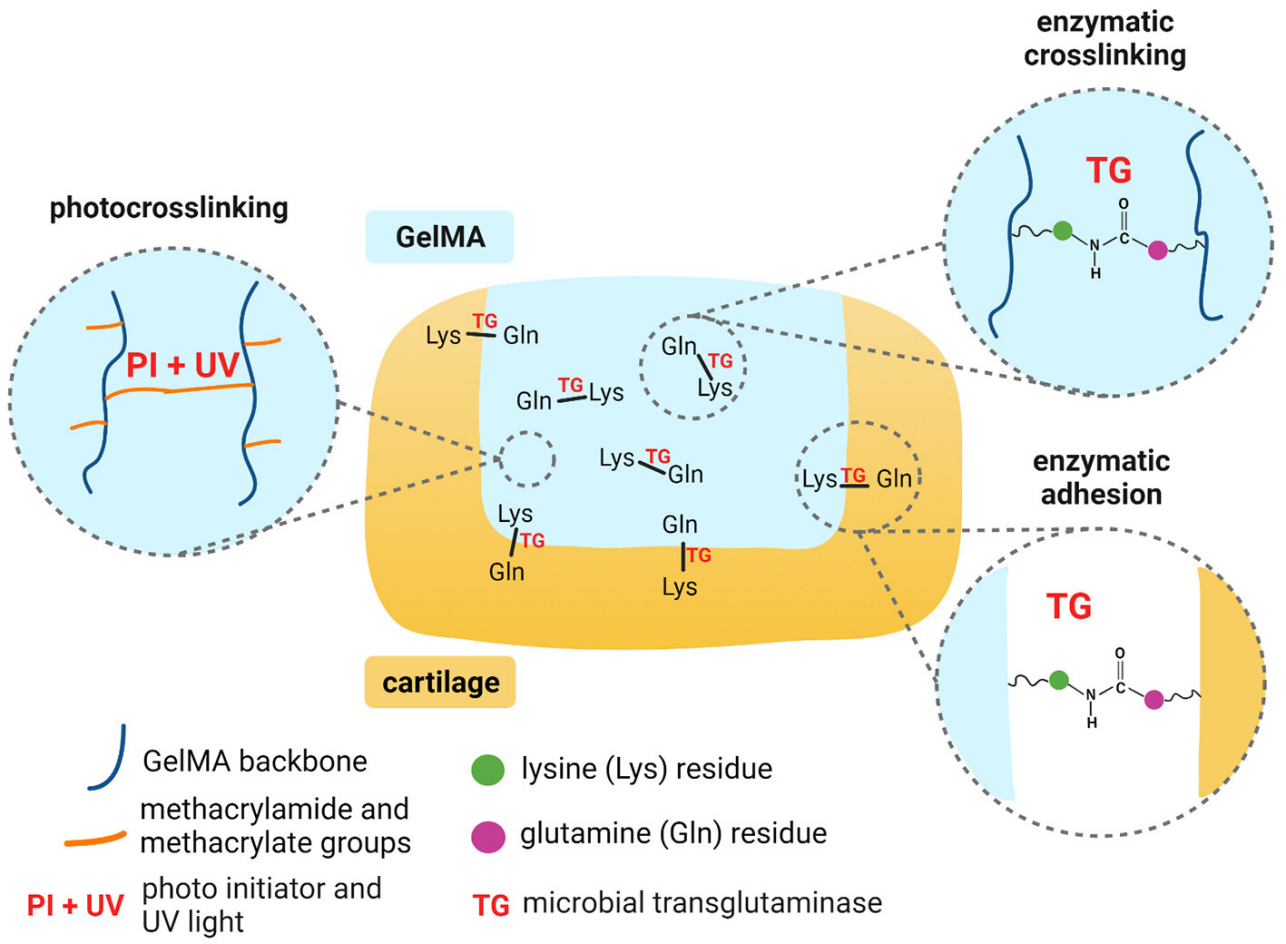
Mechanisms for dual crosslinking of GelMA and enzymatic adhesion of GelMA to cartilage. Created with BioRender.com.

Depending on the degree of methacryloyl functionalization, GelM possesses a proportion of non-substituted lysine groups available for interaction with transglutaminase. Previous studies have shown that microbial transglutaminase can be used to modify the rheological properties of GelMA (32). The enzyme has also been used to crosslink gelatin in several applications: to encapsulate cells for enhanced differentiation; to embed cells with cartilage extracellular matrix in a scaffold for cartilage repair; and to act as a tissue binding glue (33–35). To our knowledge, no studies have yet explored GelMA together with microbial transglutaminase as an adhesive implant for chondral defects. Additionally, in studies of its use as a bioadhesive, microbial transglutaminase has typically only been tested on animal derived tissues.

This work therefore aimed to investigate the use of microbial transglutaminase with GelMA for adhesion to cartilage, combined with *in situ* photocrosslinking (Scheme 1). In the first instance, we evaluated the impact of transglutaminase on the mechanical and rheological properties of GelMA, to validate sufficient substrate and enzyme interaction. We also evaluated the effects of transglutaminase on the biodegradability of photocrosslinked GelMA scaffolds and the biocompatibility of the enzyme in terms of the metabolic activity of human adipose-derived stem cells encapsulated in the biomaterial. As a primary measure, this study evaluates the ability of microbial transglutaminase to adhere *in situ* photocrosslinked GelMA to human cartilage tissue *ex vivo*. Together, these results demonstrate the feasibility of GelMA combined with transglutaminase as a biocompatible and biodegradable hydrogel that can adhere to human cartilage.

## Materials and Methods

### Materials

Gelatin type A from porcine skin (~300 Bloom) was purchased from Sigma-Aldrich (St Louis, MO, USA), and gelatin methacryloyl (GelMA) with ~85% degree of functionalization was used, building on previous work using the same GelMA product provided by TRICEP (Wollongong, NSW, Australia) (36). Fluorescent isothiocyanate isomer I (FITC) was conjugated to GelMA as previously reported (36). GelMA, GelMA-FITC and gelatin were sterilized using ethylene oxide (EtO) gas based on our previously reported method (37). Lithium phenyl-2,4,6-trimethylbenzoylphosphinate (LAP) (Sigma-Aldrich, St Louis, MO, USA) was used as a photo initiator, prepared in 4% w/v stock solution in phosphate buffered saline (PBS) (Gibco, Thermo Fisher Scientific Inc., Waltham, MA, USA) containing 100 U/ml penicillin and 100 ug/ml of streptomycin (Gibco, Thermo Fisher Scientific Inc., Waltham, MA, USA). Microbial transglutaminase (TG) (Moo Gloo TI Transglutaminase, Modernist Pantry, Portsmouth, New Hampshire) was dissolved in PBS and filter sterilized through a 0.22 μm syringe filter. Transglutaminase enzyme activity was ~100 U/g as measured by a colorimetric hydroxamate assay (38).

### Stem Cell Isolation and Culture

Human adipose derived stem cells (hADSCs) isolated from infrapatellar fat pad were obtained from consenting patients with mild/severe osteoarthritis undergoing total knee arthroplasty. The use of human samples and procedures in this study was approved by the Human Research Ethics Committee Research Governance Unit of St. Vincent's Hospital, Melbourne, Australia [HREC/16/SVHM/186]. All experiments were performed in accordance with relevant guidelines and regulations. Cells were isolated and expanded as previously described (39).

Briefly, the fat pad was diced with a scalpel and then digested with 0.1% Collagenase type I (Worthington Biochemical Corporation, Lakewood, NJ, USA) for 3 h at 37°C under agitation. The digested fat was then filtered through a nylon 100 μm cell strainer (BD Falcon) and centrifuged at 400 g for 5 min at room temperature. Supernatant was discarded, and the cell pellet resuspended in Red Cell Lysis Buffer (Sigma Aldrich) and incubated for 10 min at room temperature. After centrifugation at 400 g for 5 min, the lysate was again filtered through a cell strainer (40 μm) (BD Falcon). The isolated cells were then plated and allowed to adhere, incubating in low glucose DMEM (Sigma-Aldrich) supplemented with 10% FBS (Gibco), 100 U/ml penicillin and 100 ug/ml streptomycin solution, 2 mM L-glutamine (Gibco), 15 mM HEPES, 20 ng/ml epidermal growth factor (EGF) and 1 ng/ml fibroblast growth factor (FGF) (R&D Systems Inc, Minneapolis, MN, USA). After 48 h incubation, non-adherent cells were removed, and the media was replaced. Cells were further expanded in the same culture media for surface epitope immunophenotypic characterization (flow cytometry) and multilineage differentiation as previously reported (40).

### Fabrication of Scaffolds

GelMA was dissolved in sterile 1 × PBS containing 100 U/ml penicillin and 100 ug/ml of streptomycin to a stock concentration of 12.5% w/v. Aliquots were prepared to achieve final GelMA concentrations of 6, 8, and 10% w/v with the remaining components of the gel. These groups were selected based on a previous study where these concentrations showed suitable properties for cartilage regeneration *in vitro* (36). Stock LAP solution was added to a final concentration of 0.05%, and for scaffolds containing transglutaminase, the final enzyme concentration was 1 U/ml. Cellular scaffolds included hADSCs to a final concentration of 6 million cells/ml. For degradation tests using acellular scaffolds, GelMA-FITC was included to a final concentration of 1% w/v. For these fluorescent scaffolds, the proportion of non-fluorescent GelMA was adjusted so the total GelMA polymer concentration (GelMA + GelMA-FITC) was held at 6, 8, and 10% w/v. Materials were well-mixed prior to use. An equal starting quantity of fluorescent material was prioritized over an equal proportion of GelMA:GelMA-FITC to ensure all scaffolds tested had the same starting amount of fluorescence at the time of fabrication. Cylindrical polydimethylsiloxane (PDMS) molds of 2 mm height × 4 mm diameter were used to control the shape of the scaffolds. Scaffolds were photocrosslinked by filling molds sandwiched between two glass cover slips, which were placed under a visible light source (BioLambda, São Paulo, Brazil) for 30 sec at 405 nm and 20 mW/cm^2^. Scaffolds were carefully removed, briefly washed in PBS to remove uncrosslinked material, and incubated in PBS (acellular scaffolds) or media (cellular scaffolds) at 37°C. All mechanical tests were conducted after overnight incubation. For a summary of samples prepared at different conditions and tests performed on these samples, refer to Table 1.

**Table 1:** Summary of samples prepared at different conditions.

**Test**	**GelMA concentration (w/v)**	**TG content**
Rheology, swelling, ratio	10, 8, and 6%	–, +
Metabolic, activity, lap shear	10%	–, +
Compression, push out, degradation	10, 8, 7, and 6 %	–, +

*Note for the degradation studies, the total GelMA concentration included 1% w/v fluorescently labeled GelMA. Additionally, 7% GelMA was tested with transglutaminase only, as it was selected to match the stiffness of 8% GelMA without transglutaminase (as later described)*.

### Rheological Testing

Rheology was assessed on a Physica MCR 302 Rheometer (AntonParr) with a cone-plate geometry (15 mm diameter with angle of 1°) at 37°C. The apparent viscosity of GelMA with transglutaminase was measured after 5 min incubation at 37°C, and subsequently at 40 min intervals (incubating at 37°C between measurements) to monitor the effect of enzymatic crosslinking. The viscosity was measured at a shear rate of 100 s^−1^ for 1 min. Measurements were performed in triplicate for each condition, and for the GelMA controls at the initial time point and final time points. The flow behavior was also measured following incubation at 37°C at the same time intervals, with a shear rate ramping from 0.1 to 1,000 s^−1^ over 200 s. A moving average filter was applied to the viscosity as a function of shear rate data with a window size of seven data points.

### Mechanical Testing of Scaffolds

After overnight incubation in PBS at 37°C, photocrosslinked scaffolds were removed from the incubator to reach room temperature for 30 min. Microscopy images were taken prior to testing to allow for calculation of the cross-sectional area of each sample using ImageJ software (NIH, USA). Mechanical tests were performed at room temperature on a TA ElectroForce 5500 mechanical testing device (TA Instruments, New Castle, DE, USA) fitted with a 250 g load cell. Scaffolds were placed on a glass slide and loaded between two compression platens. Samples underwent unconfined compression at a strain rate of 0.01 mm/s up to 20–25% total displacement. A custom Python program was used to calculate the height of each sample *via* the inflection point of the load-displacement curve. The raw load-displacement data was then converted to engineering stress and strain, and used to calculate the compressive modulus as the slope of the stress-strain curve over the range of 10% to 15% strain for each sample, as previously reported (41).

### Enzymatic Degradation of Scaffolds

A previously validated method was used to track the enzymatic degradation of the hydrogel using fluorescence (36). In brief, after overnight incubation and swelling, PBS was removed and replaced with 1 ml PBS containing collagenase type II at a concentration of 5 U/ml (Worthington Biochemical Corporation) to commence enzymatic degradation upon incubation at 37°C. For the first 9 h of the experiment, collections of the PBS containing degraded fluorescent material were made every 90 min, and PBS/collagenase was refreshed. Collected PBS solution was stored at −80°C. After refreshing PBS/collagenase, fluorescence images of each condition were taken using a ChemiDoc imaging system (Bio-Rad, Hercules, CA, USA) before returning the samples to 37°C. After 9 h, collections of PBS and images were taken once per day until no scaffold remained. A set of scaffolds were also left in the original PBS/collagenase solution, without collections or refreshing, until completely degraded. Once completely degraded, this PBS containing the fully degraded scaffolds was collected to be used as a measure of the maximum amount of fluorescence. PBS collections were thawed at room temperature before being dispensed in a 96 well plate in triplicate to be measured. Fluorescence of the solution was measured using a CLARIOstar plate reader (BMG LABTECH, Freiburg, Germany) at 483–14 nm excitation and 530–30 nm emission wavelengths using the same gain for all readings. The cumulative loss was plotted as the residual percentage of material with a triplicate for each condition.

### Swelling Ratio

Scaffolds were prepared as previously described and weighed following overnight swelling (w_s_). Scaffolds were rinsed in MilliQ water, and then freeze-dried until all water was removed. After freeze-drying, all scaffolds were weighed (w_d_) and the swelling ratio was calculated by Equation 1.


(1) $$\begin{array}{r} \text{Swelling ratio}=\left({\text{w}}_{\text{s}}-{\text{w}}_{\text{d}}\right)/{\text{w}}_{\text{d}} \end{array}$$

### Metabolic Activity Assay

Cellular and acellular scaffolds of 10% GelMA were cast and photocrosslinked, both with and without transglutaminase as described above. After photocrosslinking, scaffolds were incubated in culture media containing low glucose DMEM (Sigma-Aldrich) supplemented with 10% FBS (Gibco), 100 U/ml penicillin and 100 ug/ml streptomycin (Gibco), 2 mM L-glutamine (Gibco), and 15 mM HEPES (Gibco), 20 ng/ml epidermal growth factor (EGF) and 1 ng/ml fibroblast growth factor (FGF) (R&D Systems Inc, Minneapolis, MN, USA). At days 1, 3, and 7, a biological triplicate of each condition was removed to perform a metabolic activity assay using CellTiter-Blue® Reagent (Promega, Madison, WI, USA) according to manufacturer's instructions and based on a previously published method which assessed metabolic activity of cells in 3D (42). Scaffolds were incubated for 3 h in a 1:5 ratio of media and reagent. After incubation, supernatant was collected and measured using a CLARIOstar plate reader at 550–15 nm excitation and 600–20 nm emission using the same gain for all readings. After the metabolic assay, scaffolds were rinsed in PBS before performing a mechanical unconfined compression test as described.

### Quantification of Adhesion

One of the difficulties of assessing bioadhesives is reliably quantifying the strength of adhesion to tissue under conditions similar to those experienced in the body (hydrated tissue, 37°C). Here we have used two methods to quantify adhesion. A lap shear test based on ASTM standard F2255–05 against bovine articular cartilage (supplied fresh by a local butcher), and a push out test using human cartilage explants (43). Human cartilage samples were obtained from consenting patients with mild/severe osteoarthritis undergoing total knee arthroplasty. The use of human samples and procedures in this study was approved by the Human Research Ethics Committee Research Governance Unit of St. Vincent's Hospital, Melbourne, Australia [HREC/16/SVHM/186]. All experiments were performed in accordance with relevant guidelines and regulations.

### Lap Shear Test

**Figure 1:**
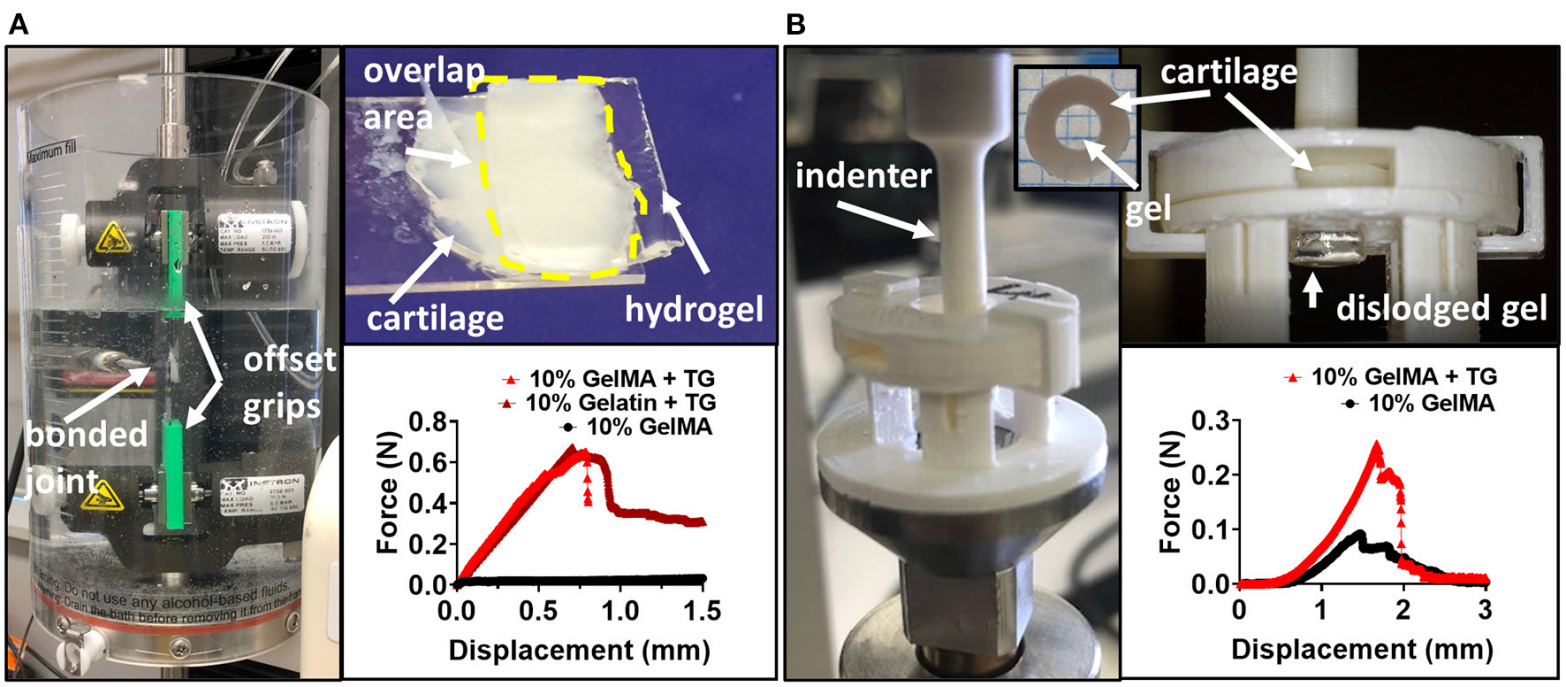
Testing the adhesive ability of GelMA with transglutaminase hydrogels against cartilage tissue **(A)** Pictures depicting lap shear test (left and top) and representative force-displacement curves for tests. **(B)** Pictures depicting *ex vivo* model and push out test (left and top), and representative force-displacement curves for tests with 10% GelMA. For representative force-displacement curves for 8 and 6% GelMA, see Supplementary Figure 5.

Cartilage was removed from bovine stifle joints with a scalpel and placed in PBS containing penicillin-streptomycin (100 U/ml−100 ug/ml) and fungin (0.01 mg/ml, InvivoGen, San Diego, CA, USA). GelMA biomaterials with and without TG were prepared as previously described, and gelatin was prepared to a concentration of 10% w/v in PBS with 1 U/ml TG. For GelMA (with and without TG), material was cast directly onto the cartilage surface, with liquid contained by a PDMS mold (ranging from 5 to 10 mm in width, and 15–25 mm in length, depending on the cartilage sample size), and then photocrosslinked with visible light under the conditions previously described. For the gelatin condition, a PDMS mold was placed on a glass slide and filled with the gelatin and TG solution and allowed to thermally gel at room temperature. Cartilage of similar dimensions (5–15 mm width, 15–25 mm length) was then placed on the surface of the gel. All samples were then placed in a custom silicone chamber designed to create sterile, humid conditions at 37°C for the samples to swell and adhere overnight (Supplementary Figure 1). The chamber set up was designed to ensure an offset and even pressure on the tissue-gel bond, with a weight placed on top of the bond applying a ~0.8 N force throughout curing. After swelling, samples were tested using custom 3D-printed polylactic acid (PLA) grips designed to ensure the uniaxial tensile force was applied in alignment with the bonded surface. The test was performed using an Instron 5944 microtester (Instron, Norwood, MA, US) fitted with a 50 N load cell. Samples were tested hydrated and swollen in a 37°C water bath containing PBS. The tensile test was performed at a strain rate of 5 mm/s in accordance with ASTM standard F2255–05 (2015). Prior to testing, images were taken with a smartphone camera to calculate the bond overlap area using ImageJ (note the image in Figure 1A is for illustration of the cartilage and hydrogel only, and images for calculating the area were taken directly above the sample with a scale bar included). The maximum force at failure of the bond was normalized by the bond overlap area to determine the lap shear strength (43).

### Push Out Test

Disks of cartilage were cut from the surface of human condyles using an 8 mm biopsy punch and a scalpel and stored in PBS containing penicillin-streptomycin and fungin. Defects were created in each cartilage disk using a 4 mm biopsy punch. GelMA (with and without TG) was cast into the cartilage defects, and photocrosslinked with visible light. A PDMS stage and barriers were used to avoid leakage of the material from the bottom of the cartilage ring, or overflow on top of the ring. After photocrosslinking, samples were submerged in 1.5 ml PBS, and incubated overnight at 37°C. On the day of testing, samples were removed from the incubator to acclimatize to room temperature for a minimum of 30 min prior to testing. Testing was performed at room temperature on a TA ElectroForce 5500 mechanical testing device fitted with a 250 g load cell, with tissue kept hydrated during testing with drops of PBS. Before performing the push out test, an indentation test was performed using a 1 mm diameter stainless steel cylindrical indenter. Indentation was performed to a strain depth of no >10%, allowing for accurate calculation of the height of the scaffold within the cartilage ring by identifying the point of contact between the indenter and the top of the scaffold. This indentation was performed at a strain rate of 0.01 mm/s and a custom Python program was used to identify the inflection in the load-displacement curve indicating contact with the top of the scaffold. After this test, samples were immediately returned to PBS to stop the tissue drying out. The push out test was performed using custom 3D printed PLA indenters of 3.4–3.8 mm diameter to dislodge the gel scaffold from the cartilage ring. Samples were held securely in place using a 3D printed stage and lid to prevent lateral and vertical movement of the cartilage ring (Figure 1B). The contact area between the gel and the inner surface of the cartilage defect was calculated as the circumference of the cartilage defect multiplied by the height of the scaffold. The maximum force to dislodge the sample was normalized by the contact area to calculate the push out strength.

### Statistical Analysis

Statistical analyses were performed using Prism 8 (GraphPad Software Inc.). Analysis of variance (ANOVA) was used to test for statistical significance between multiple groups with Bonferroni *post-hoc* tests, and a statistical significance level of 0.05. Unpaired *t*-tests were performed to compare two groups with Bonferroni correction.

## Results

### Rheological and Mechanical Analysis of GelMA With Transglutaminase

**Figure 2:**
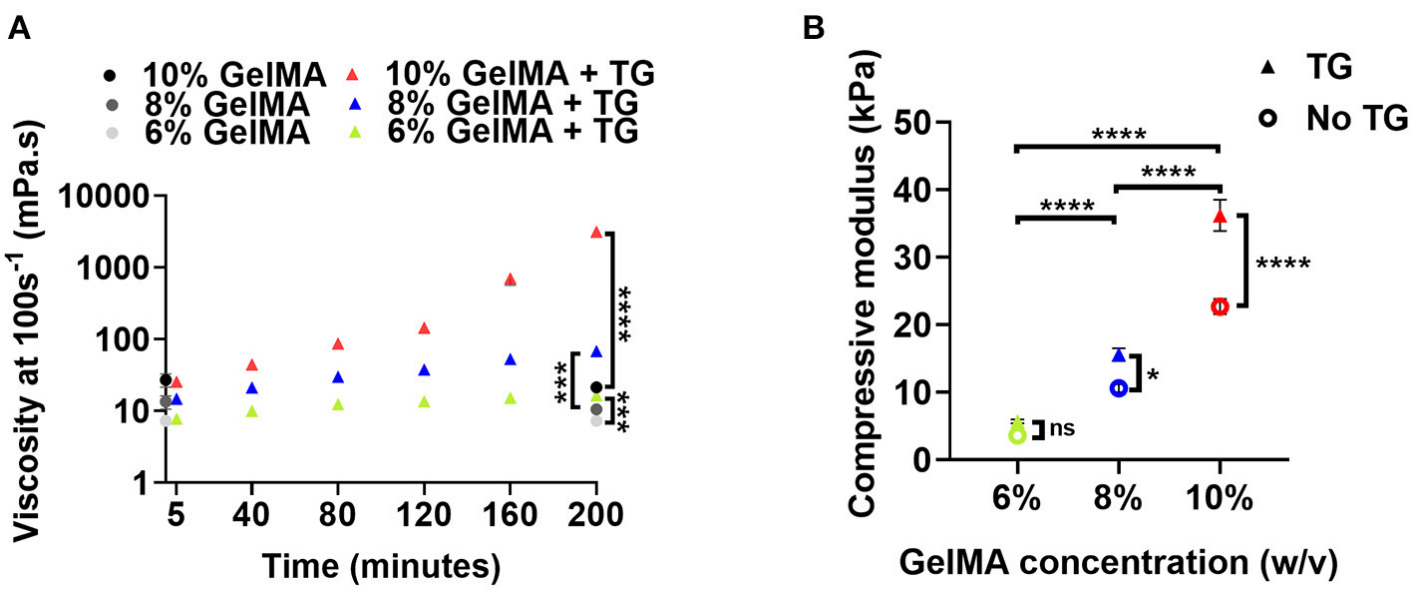
Material properties of GelMA combined with transglutaminase at different substrate concentrations **(A)** Viscosity at a shear rate of 100 s^−1^ of three concentrations of GelMA incubated with transglutaminase at 37°C. Viscosity is first plotted at 5 min, and then at intervals of 40 min up to 200 min. Data points represent the means of measurements in triplicate with 60 data points averaged for each measurement and error bars show the standard deviation. Controls without transglutaminase are shown at 0 min and the final time point only. Significance at 200 min was assessed using unpaired *t*-tests with Bonferroni correction. ^***^p < 0.001, ^****^p < 0.0001. **(B)** Unconfined mechanical compression analysis of GelMA scaffolds at three concentrations, with and without transglutaminase, after overnight incubation at 37 °C plotted as compressive modulus. Data points are means and error bars show standard deviations (*n* = 3 or 4). Significance was assessed using a two-way ANOVA with Bonferroni *post-hoc* test. ^*^p < 0.05, ^****^p < 0.0001.

The viscosity of GelMA combined with transglutaminase was measured *via* rheology at a shear rate of 100 s^−1^ after different stages of incubation at 37°C to evaluate the time-dependent impact of transglutaminase on the material in the absence of photocrosslinking (Figure 2A). After addition of transglutaminase and 5 min incubation at 37°C to ensure a homogeneous solution, no significant difference between GelMA with transglutaminase and controls at the same polymer concentrations (at time point 0 min) was observed (unpaired *t*-tests with Bonferroni correction).

The viscosity for all GelMA concentrations was observed to increase with incubation time. For all concentrations, this increase in viscosity was significant after the first 40 min of incubation (repeated measures ANOVA, Bonferroni *post-hoc* test). Though all materials undergo a significant increase in viscosity over 200 min compared to controls, this only holds practical significance for 10% GelMA, which sees a sharp increase in viscosity after 160 min incubation (~145 mPa.s to 705 mPa.s) as the material begins to form a weak gel. A yield stress was also observed after 200 min incubation (Supplementary Figure 2D), indicative of the formation of microstructure due to transglutaminase-induced crosslinking. Conversely, after more than 3 h incubation at 37°C, the viscosities of 8% and 6% GelMA remained below 0.2 Pa.s over a shear rate ramp (Supplementary Figures 2E–F). Based on a simple inversion test, 6% GelMA and 8% GelMA combined with transglutaminase do not appear to gel after 24 h incubation (data not shown).

The mechanical properties of GelMA combined with transglutaminase after photocrosslinking were assessed by calculating the compressive modulus. The results for different concentrations of GelMA with and without transglutaminase are plotted in Figure 2B. To generate GelMA + TG scaffolds for compression tests, transglutaminase was added to the biomaterial prior to photocrosslinking with visible light. After photocrosslinking, scaffolds were incubated in PBS overnight in order to swell and allow for enzymatic crosslinking.

The addition of transglutaminase saw a trend of increased compressive modulus at each GelMA concentration. A two-way ANOVA showed that both transglutaminase and the concentration of GelMA had a significant effect on the stiffness of the scaffold, increasing compressive modulus with polymer concentration and addition of transglutaminase (*p* < 0.0001). A significant interaction effect was also observed (*p* < 0.0001), with the effect of transglutaminase amplified at higher polymer concentrations (e.g., 10% GelMA + TG = 38.1 kPa ± 3.4 was significantly >10% GelMA = 22.7 kPa ± 2.3 by a *post-hoc* Bonferroni test, *p* < 0.0001).

The swelling ratio of scaffolds was also evaluated, with the results showing a trend of decreasing swelling ratio with increasing concentration of GelMA (Supplementary Figure 3). The addition of transglutaminase however, did not have a significant effect on the swelling ratio.

### Enzymatic Degradation Behavior of GelMA Scaffolds Containing Transglutaminase

**Figure 3:**
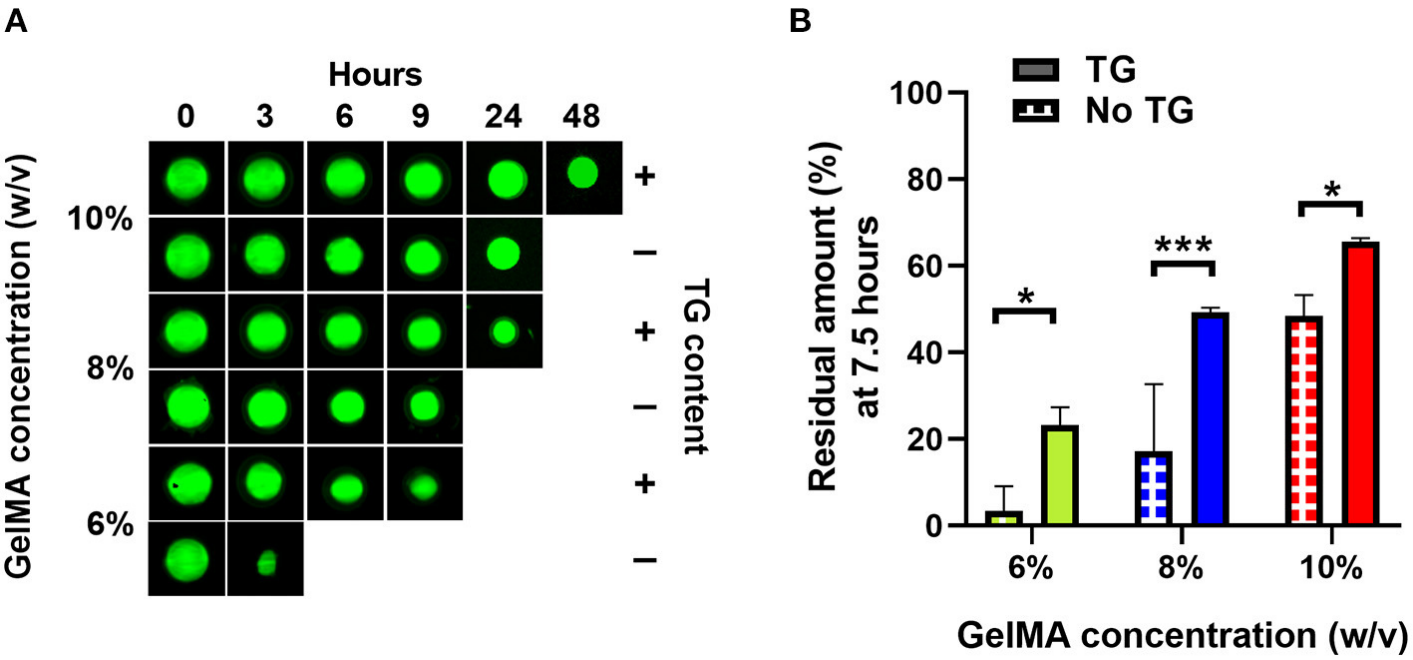
Enzymatic degradation of GelMA with and without transglutaminase at different substrate concentrations. **(A)** Fluorescence imaging of GelMA scaffolds at 10, 8, and 6% concentrations, without and with transglutaminase after incubation for different times in PBS containing collagenase type II (5 U/ml). **(B)** Residual amounts of scaffolds remaining after 7.5 h incubation calculated *via* cumulative loss of fluorescent material for GelMA scaffolds at three concentrations, both without and with transglutaminase. Data points are means with standard deviation for three scaffolds for each condition. Significance was assessed using a two-way ANOVA with Bonferroni *post-hoc* test. ^*^p < 0.05, ^***^p < 0.001.

Using a previously reported method, we employed fluorescent material to track the degradation of GelMA scaffolds containing transglutaminase with the enzyme collagenase type II added to the PBS for biologically mediated degradation (36). Fluorescent images of individual scaffolds illustrate that those containing transglutaminase remained stable in the enzymatic solution for longer relative to controls at the same GelMA concentration (Figure 3A).

The same trend is observed quantitatively, where the addition of transglutaminase to the GelMA scaffolds led to an increase in the time required to fully degrade the material based on the residual amount of material, which was calculated using fluorescence readings of degraded material released into solution at each time point (Supplementary Figure 4).

As in Figure 3B, after 7.5 h degradation, there were significantly greater amounts of all scaffolds containing transglutaminase relative to their controls (6% GelMA: 23.3 ± 4.0% compared to 3.3 ± 5.7%, *p* = 0.017, 8% GelMA: 49.3 ± 1.1% compared to 17.1 ± 15.5%, *p* = 0.0005, 10% GelMA: 65.6 ± 1.0% compared to 48.5 ± 4.8%, *p* = 0.041).

Interestingly, 8% GelMA + TG degraded at a similar rate to 10% GelMA over the first 9 h (and comparable amounts of scaffold remained after 7.5 h), despite 10% GelMA having significantly higher compressive modulus than 8% GelMA +TG (Figure 2B).

### Biocompatibility of Transglutaminase

**Figure 4:**
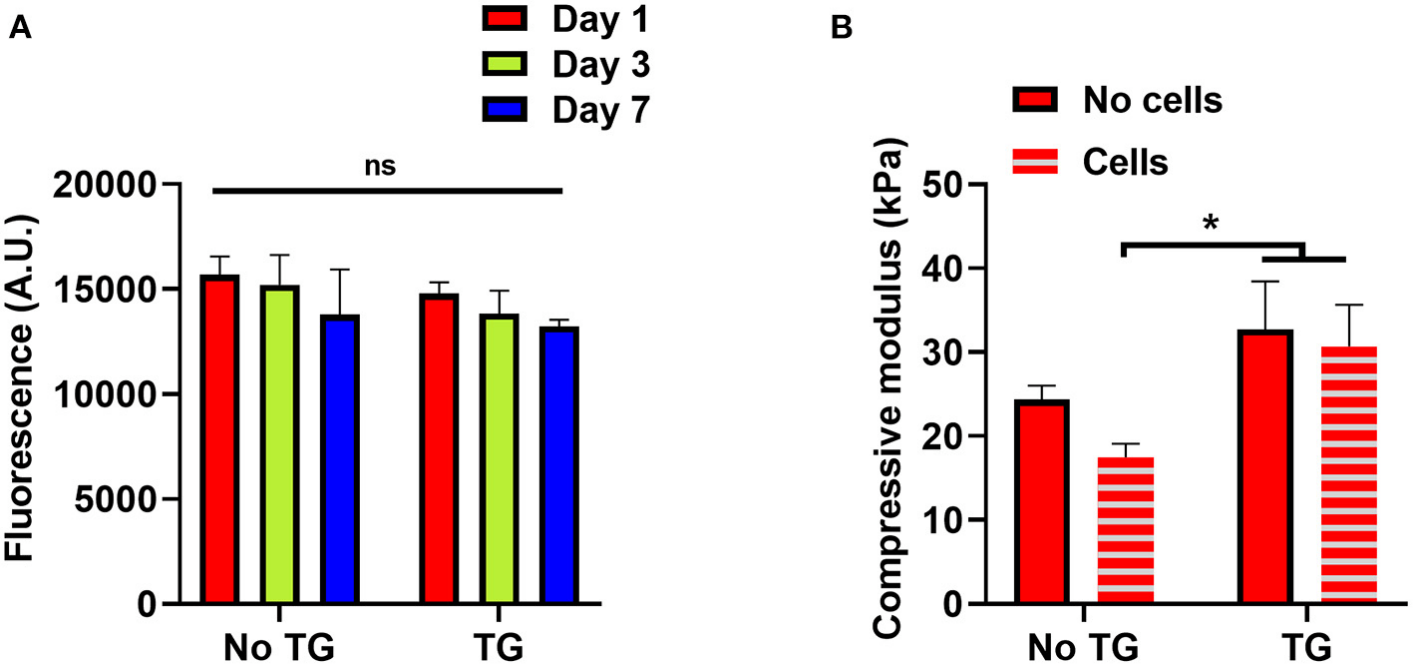
Reciprocal effects of transglutaminase and hADSCs on metabolic and enzymatic activity **(A)** Cell titer blue assay results to quantify the metabolic activity of hADSCs encapsulated in 10% GelMA scaffolds, with and without transglutaminase, over 7 days. Bars show means with standard deviation for three samples at each timepoint for each condition. Statistical analysis was performed by two-way ANOVA with Bonferroni *post-hoc* test. **(B)** Unconfined mechanical compression analysis of cellular and acellular 10% GelMA scaffolds, with and without transglutaminase, at day 1. Each bar shows mean with standard deviation for three samples. Statistical significance was assessed by two-way ANOVA with Bonferroni *post-hoc* test. ^*^p < 0.05.

To investigate the impact of transglutaminase on hADSCs, cellular scaffolds were cast using 10% GelMA with and without transglutaminase. Of the conditions used earlier, 10% GelMA was chosen to provide the largest quantity of substrate to interact with the enzyme, and observe the impact of transglutaminase induced crosslinking on encapsulated cells. The scaffolds were maintained in culture for 7 days. Over the 7 day period, a slight downward trend in fluorescence units as a measure of metabolic activity of live cells contained within the scaffolds was observed for both conditions, with and without transglutaminase, but overall no significant changes were observed (Figure 4A).

To assess whether the cells had any impact on the activity of transglutaminase, unconfined compression tests were also performed on these scaffolds, using the modulus of the material as an indicator of the ability for transglutaminase to form covalent crosslinks. There was no significant difference in the compressive modulus of the GelMA + TG condition between cellular and acellular scaffolds, whereas a decrease was seen in the GelMA controls containing cells. Indeed, the compressive modulus of the scaffolds containing transglutaminase was significantly larger at day 1 than that for cellular scaffolds without transglutaminase (Figure 4B).

### Quantification of Adhesion of GelMA Hydrogels Containing Transglutaminase to Cartilage

A lap shear test, commonly used in the literature, was performed to assess adhesion of the biomaterial against bovine cartilage. Under a lap shear test configuration, GelMA alone could not adhere to the tissue, with few samples able to be loaded into the testing device without the sample visibly slipping on the surface of the cartilage during the process. Those that were loaded did not exhibit a clear failure of the bond, but rather a low force due to friction, as the two surfaces slid across each other (Figure 1A).

**Figure 5:**
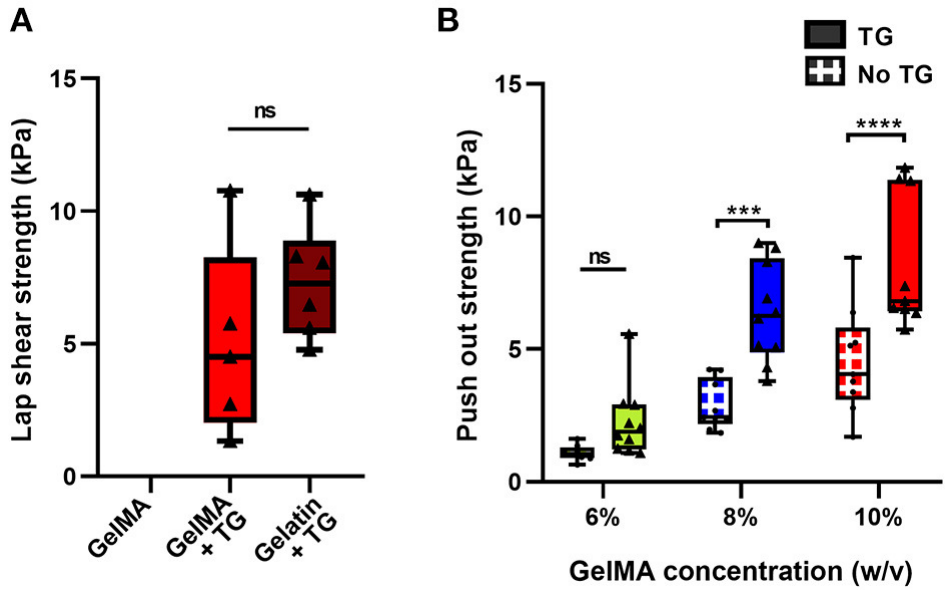
Quantification of the adhesive ability of GelMA with transglutaminase against cartilage tissue **(A)** Lap shear analysis of 10% GelMA with transglutaminase against bovine cartilage, in comparison to GelMA only and 10% gelatin with transglutaminase as controls. Individual data points are plotted with box and whisker plots indicating the median and interquartile ranges. Significance was assessed with an unpaired *t*-test. **(B)** Push out test results for three concentrations of GelMA with and without transglutaminase. Individual data points are plotted with box and whisker plots indicating the median and interquartile ranges. Significance was assessed with two-way ANOVA and Bonferroni *post-hoc* test. ^***^p < 0.001, ^****^p < 0.0001. Triangle symbols and solid shaded boxes are used to indicate samples that contained transglutaminase, and circle symbols with hatched boxes are used to indicate controls.

The addition of transglutaminase resulted in adhesion of the photocrosslinked 10% GelMA to the surface of the cartilage, with a mean lap shear strength of 5.0 kPa ± 3.6 (Figure 5A). As a positive control for comparison, gelatin at 10% w/v crosslinked by transglutaminase was also bonded to the surface of cartilage to measure adhesion. This resulted in a mean lap shear strength of 7.3 kPa ± 2.1, with no significant difference to 10% GelMA + TG.

An *ex vivo* model of cartilage repair was used to assess adhesion to human tissue. Disks of cartilage were cut from human condyle explants, and a defect was created in each disk and filled with the bioadhesive material (Figure 1B). Using a push out test, the addition of transglutaminase to GelMA resulted in a trend of increasing push out strength with increasing concentrations of GelMA (Figure 5B). Based on two-way ANOVA, 10% GelMA + TG and 8% GelMA + TG were both significantly higher than their respective controls (8.2 kPa ± 2.5 compared to 4.5 kPa ± 2.0, *p* < 0.0001 and 6.4 kPa ± 1.9 compared to 2.9 kPa ± 0.9, *p* = 0.0001, respectively), whilst there was no significant difference between 6% GelMA + TG and its control.

### Adhesion and Degradation Analysis of Samples With Matched Stiffness

**Figure 6:**
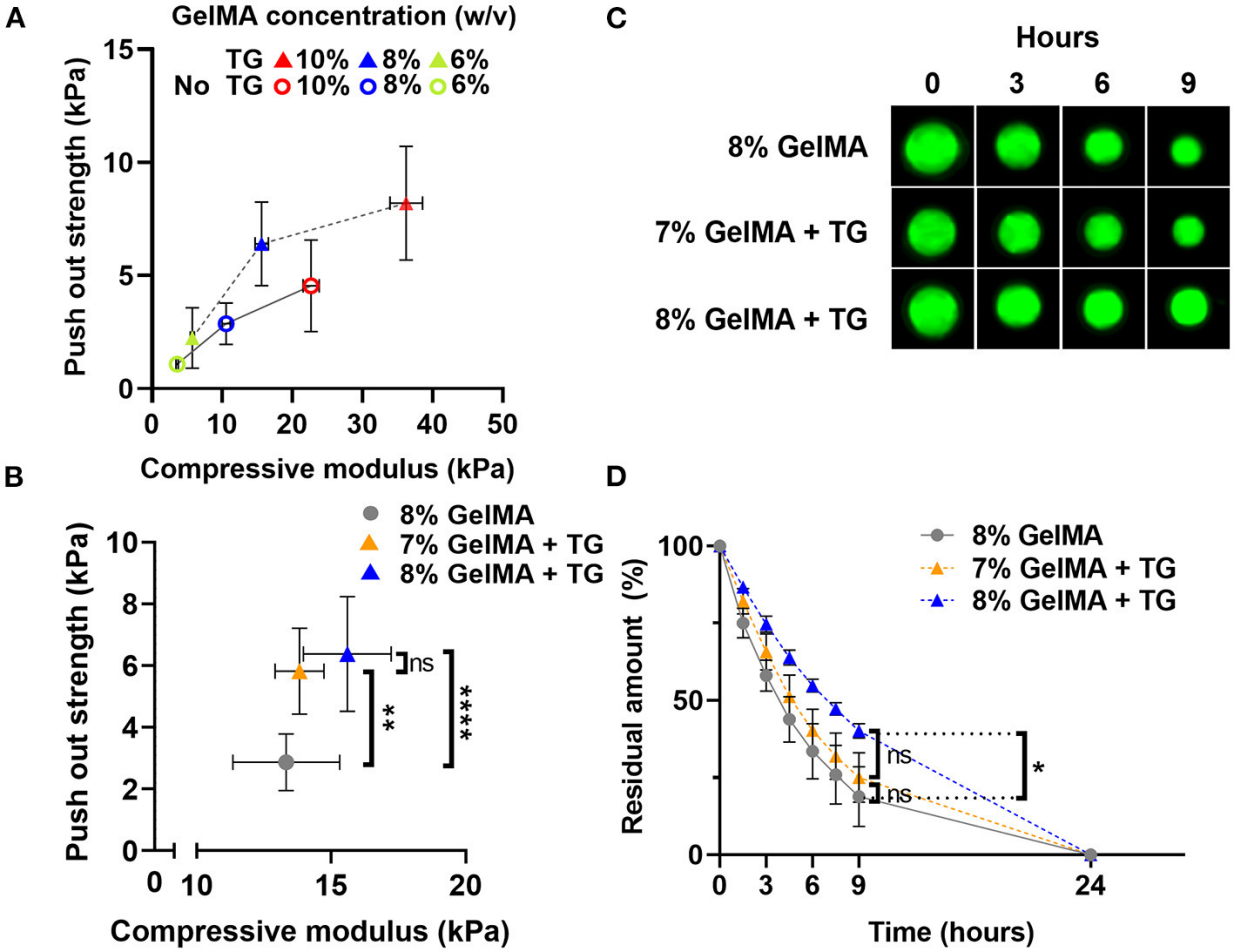
Comparison of push out strength and enzymatic degradation performance for materials with matched stiffness **(A)** Scatter plot of push out strength against compressive modulus (kPa) for three GelMA concentrations with and without transglutaminase. **(B)** Scatter plot of push out strength against compressive modulus for 8% GelMA without and with transglutaminase, and 7% GelMA with transglutaminase. Data points represent mean and standard deviation of three samples for compression, and six or more samples for push out test. Significance was assessed *via* one-way ANOVA with Bonferroni *post-hoc* test. ^**^p < 0.01, ^****^p < 0.0001. **(C)** Fluorescence imaging of GelMA scaffolds at 8%, with and without transglutaminase, and 7% with transglutaminase after incubation in PBS containing collagenase type II (5 U/ml). **(D)** Residual amount of scaffold remaining calculated *via* cumulative loss of fluorescent material for GelMA scaffolds at 8%, with and without transglutaminase, and 7% with transglutaminase. Data points are the mean with standard deviation for three scaffolds for each condition. Significance was assessed using a one-way ANOVA with Bonferroni *post-hoc* test. ^*^p < 0.05.

Illustrated in Figure 6A, the push out strength increased with increases in the bulk strength (compressive modulus) of the material, both for samples without and with transglutaminase. Overall, samples with transglutaminase tended to have higher push out strengths than those without.

To decouple the effect of the bulk strength of the material from the push out strength as a measure of adhesion, samples of matched stiffness with and without transglutaminase were analyzed. Samples of 7% GelMA with transglutaminase were found to have comparable compressive modulus to 8% GelMA samples without transglutaminase (Figure 6B, 13.8 kPa ± 0.9 and 13.3 kPa ± 2.0, respectively, *p* > 0.999). At these polymer concentrations, push out strength was found to significantly increase in the presence of transglutaminase despite their matching bulk mechanical properties (Figure 6B, 7% GelMA + TG: 5.8 kPa ± 1.4, 8% GelMA: 2.9 kPa ± 0.9, *p* = 0.003). This provides evidence in support of the hypothesis that transglutaminase can form covalent bonds between GelMA and the surface of the cartilage, rather than simply increasing the bulk strength of the gel and its resistance to being dislodged by the indenter.

Fluorescent scaffolds incubated in PBS containing collagenase type II (5 U/ml) are shown in Figure 6C, qualitatively illustrating similar behavior between the 8% GelMA and 7% GelMA with transglutaminase samples. After 9 h, both are reduced in size in comparison to the 8% GelMA with transglutaminase condition. The quantitative results of this experiment are plotted in Figure 6D, showing the residual amount of fluorescent material remaining at each time point. After 9 h incubation in collagenase type II, there is no significant difference between 8 and 7% GelMA with transglutaminase by one-way ANOVA with Bonferroni *post-hoc* test (*p* > 0.9999). This demonstrates the comparable biodegradability of these scaffolds with matched stiffness, regardless of the transglutaminase content.

## Discussion

Untreated, damaged articular cartilage can result in post-traumatic osteoarthritis (7). Current surgical techniques to treat cartilage defects do not have satisfactory long-term results, and though tissue engineering approaches show promise, their long-term outcomes remain to be seen (9, 11). The failure to laterally integrate grafts, biomaterials or newly generated cartilage with the native tissue is thought to be one of the recurring reasons preventing long-term repair (15, 44). A further difficulty in designing cartilage treatment strategies that integrate well with native cartilage, is simply evaluating the degree of integration. Few *in vitro* and *in vivo* animal studies quantify the degree of integration in terms of mechanical strength (16). Many studies are limited to qualitative scoring and histological analysis, which may not correlate with mechanically integrated tissue (45).

There is a need for a regenerative approach to cartilage repair that ensures the scaffold is well secured in the cartilage defect in the short term and supports integration in the long term. Bioadhesives are one possible strategy to overcome the challenge of integration, and have been developed as an adhesive layer that can be applied prior to implantation, a sealant that is applied after, or as an overall bioadhesive material that can deliver cells to the defect (29, 46–48).

This study proposes the addition of microbial transglutaminase to GelMA for adhesion to cartilage followed by photocrosslinking by visible light (405 nm) with a photo initiator (LAP). GelMA has previously been used as an effective matrix for human adipose derived stem cells to generate neocartilage *in vitro*, in an *ex vivo* human chondral defect model, and in an *in vivo* rabbit chondral defect model with rabbit MSCs (36).

### Transglutaminase Addition to GelMA Enables Dual Crosslinked Hydrogels With Improved Mechanical Properties

The initial hypothesis of this work was that microbial transglutaminase can catalyze covalent crosslinks between GelMA chains, utilizing remaining un-methacrylated lysine groups to interact with transglutaminase. The enzyme catalyzes an acyl transfer reaction between glutamine (with the γ-carboxyamide group as the acyl donor) and lysine (with the ε-amino group as the acyl receptor) residues found in proteins, forming an ε-(γ-glutamyl)lysine (GL) bond (30). Sufficient remaining lysine residues to interact with transglutaminase are necessary in order for the biomaterial to bond to the surface of cartilage tissue well. As a test of this hypothesis, we analyzed the impact of transglutaminase on the bulk mechanical properties of the material. We performed unconfined compression tests of uniform GelMA scaffolds containing transglutaminase, and found there was a significant increase in the compressive modulus with the addition of the enzyme. This indicated that there are sufficient groups available in this 85% functionalized GelMA for transglutaminase to react with and provide additional crosslinking. Importantly, this data also demonstrated that the visible light photocrosslinking reaction and enzymatic crosslinking reaction do not strongly interfere with one another.

This was further supported by rheological analysis showing changes in the viscosity of GelMA after incubation with transglutaminase, in the absence of photocrosslinking. The results demonstrated that the addition of transglutaminase modulates the viscosity of the material, likely through connection of GelMA molecules. Only the highest concentration of GelMA used (10%) reached a percolation threshold, suggesting the formation of a transglutaminase mediated network. After 200 min a yield stress was observed, indicated by the viscosity peak at ~320Pa.s (Supplementary Figure 2D). This yield stress suggests the formation of microstructure which is disrupted before the material begins to flow.

In a study utilizing microbial transglutaminase to modify GelMA printability with a lower degree of functionalization (~65%), the highest concentration of microbial transglutaminase (5 U/ml) saw GelMA 10% chemically gelled after ~2 h (32). In this study, weak gelation of GelMA 10% with a transglutaminase concentration of 1 U/ml was observed after a comparable time of ~3 h incubation at 37°C.

After more than 3 h of incubation at 37°C, low viscosities were still observed for 8 and 6% GelMA. However, after only 40 min the viscosity at 100 s^−1^ was seen to significantly increase above that of controls for all GelMA concentrations, suggesting that some crosslinking induced by transglutaminase had commenced. This provides a useful indicator of the window of time required for adhesion to commence (in the order of minutes and hours, not seconds). The rheological data also gives a practical understanding of the working time of the material once mixed at 37°C—for 10% GelMA evidence of irreversible chemical bonds occurs after 3 h incubation, whilst lower GelMA concentrations do not appear to have the same limitation. Whilst adhesion does not occur instantaneously, the material can be secured in the defect in the first instance by photocrosslinking (28).

### A Slower Degradation Rate Is Observed With the Addition of Transglutaminase

A study of the material under enzymatic degradation with collagenase type II demonstrates an increase in stability due to the addition of transglutaminase. Our previous work has demonstrated a relationship between the degradation rate of GelMA scaffolds (which in turn is related to its network density) and the production of hyaline-like cartilage by stem cells encapsulated in the material and undergoing chondrogenesis (36). At lower network densities (i.e., lower concentrations of GelMA), a higher production of collagen type II was observed with a larger gain in stiffness of the scaffold due to the production of cartilage matrix over a 28 day period (36). In this study, despite significantly lower compressive modulus, 8% GelMA + TG scaffolds had comparable residual amounts remaining to 10% GelMA scaffolds after 7.5 h (Figure 3B) and degraded at a similar rate over the first 9 h (Supplementary Figure 4). The addition of transglutaminase appears to have given additional stability to the scaffold under enzymatic degradation (likely due to the formation of a secondary network of covalent crosslinks), matching that of a higher polymer concentration without transglutaminase. This quality could be further investigated to optimize the mechanical and degradation properties of the material to support neocartilage formation when seeded with stem cells undergoing chondrogenesis. Beyond the concentration of the enzyme itself, transglutaminase crosslinking can be modulated *via* substrate (GelMA) concentration and by extension the degree of functionalization of the GelMA (e.g. a lower degree of functionalization will provide more lysine groups available for transglutaminase). These factors also modulate the degree of photocrosslinking of the GelMA network, in addition to the concentration of photo initiator, the intensity of the light source and duration of exposure (49).

### Transglutaminase Did Not Negatively Impact Embedded hADSCs, nor Did Cells Prevent Enzymatic Crosslinking

Microbial transglutaminase is classified as “generally recognized as safe” by the FDA and is used in food processing (30). In this study, the addition of microbial transglutaminase did not negatively impact the metabolic activity of hADSCs encapsulated in photocrosslinked GelMA scaffolds in comparison to transglutaminase free controls over a 7 day period. No increase in metabolic activity (indicating proliferation) was observed; however, previous work has shown little growth in metabolic activity of hADSCs in 10% GelMA over a 7 day period (36). It may be that 10% GelMA scaffolds have relatively poor diffusive properties, which may limit nutrient access within the scaffold.

Other studies have also demonstrated the cytocompatibility of microbial transglutaminase, with ADSCs encapsulated in gelatin crosslinked by microbial transglutaminase (60 U/g) showing good viability after 7 days *via* live/dead staining (34). Freeze-dried gelatin sponges crosslinked by microbial transglutaminase and seeded with ADSCs have also exhibited a higher proliferation rate than gelatin sponges crosslinked *via* other methods (50). Approximately 90% cell viability was observed after 7 days when myoblasts were encapsulated in printed GelMA-transglutaminase constructs, at an enzyme concentration of 3 U/ml, three times higher than that used in this study (32).

### Adhesion to Human Cartilage *ex vivo* Is Significantly Increased by Transglutaminase

One of the challenges of developing bioadhesives is reliably quantifying the adhesion of soft gel materials to comparatively stiffer biological tissue. The hydration conditions used during curing and testing are important, as cartilage dries out quickly if left in air. Tests should be performed in hydrated conditions, by using a water bath where possible, or ensuring samples are kept hydrated prior to and during the test. This study utilized two methods of testing adhesion, the lap shear test and the push out test. Both provided useful data as to the adhesive quality of GelMA containing transglutaminase and showed an increase in both lap shear strength and push out strength against cartilage tissue with the addition of transglutaminase, respectively.

In the case of the lap shear test, no adhesion was observed with GelMA alone. Though GelMA with transglutaminase did adhere to the bovine cartilage surface, there was no significant difference observed between GelMA with transglutaminase and a positive control of gelatin crosslinked by transglutaminase. Although this test is based on an ASTM standard and commonly reported in the literature, in this instance it lacked the sensitivity to differentiate between the two conditions, noting that gelatin has many more binding groups available to interact with transglutaminase than the 85% functionalized GelMA used in this study. The distinction between these two materials is clear in other ways—gelatin mixed with microbial transglutaminase at the same concentration will begin to crosslink and form a stable gel after only 11 min incubation at 37°C (data not shown), as compared to the 3 h of incubation required for evidence of gelation of GelMA with transglutaminase.

On the other hand, the push out test appeared sensitive enough to measure differences between GelMA concentrations with and without transglutaminase. Both the concentration of GelMA and the transglutaminase content had a significant effect on the push out strength, increasing with concentration and addition of transglutaminase. However, it is important to acknowledge the role the bulk stiffness of the material plays in this test. GelMA alone either did not adhere to the surface of cartilage in the lap shear test or merely slid across the tissue, registering only a frictional drag force. In the push out test, GelMA alone did register a failure in the force profile. However, this may not indicate bonding between GelMA and cartilage, but rather the material swelling in the defect and thus resisting dislodgment. An analysis of the failure profiles shows that on average, samples containing transglutaminase tend to see a sharper drop from the peak failure force to 50% of this value. Conversely, GelMA alone samples fail at a lower peak force, and register a gradual decline in force over a longer displacement to reach the equivalent 50% value (Supplementary Figure 6). The former may be indicative of the failure of covalent bonds between the material and cartilage, while the more gradual drop in force of the GelMA samples may illustrate the role of friction as the gel is dislodged.

Controlling for the compressive modulus of the material and comparing conditions of equal stiffness (7% GelMA with transglutaminase and 8% without) saw a significant increase in push out strength with the addition of transglutaminase. Combined, the results of these adhesion tests provide evidence of genuine attachment between GelMA and cartilage due to the addition of transglutaminase and the hypothesized formation of covalent bonds between the two surfaces.

The range of bond strengths to cartilage achieved in this study are similar to those reported in the literature. As assessed *via* a push out test, a study utilizing the transglutaminase activity of Factor XIIIa (by combining blood coagulation factor XIII with thrombin) to bond hyaluronan to bovine cartilage saw strengths of ~6 kPa, and a study reporting GelMA further functionalized with tyramine to bond to equine cartilage saw strengths of ~13 kPa (relative to ~7 kPa for GelMA controls) (29, 47). Using a lap shear test, Zhang et. al achieved bond strengths to porcine cartilage in the order of 10–20 kPa with an elastin-like protein/chondroitin sulfate/carboxylate-terminate poly (ethylene glycol) adhesive (48). Commercially available fibrin based adhesives are reported to have adhesion strengths in the range of ~1–27 kPa depending on the adhesion test and tissue substrate used, making comparison of results difficult (33). One advantage of this study is the access to human cartilage explants to verify the efficacy of microbial transglutaminase to bond GelMA to clinically relevant cartilage. Whether these adhesive strengths are sufficient to secure the implant *in vivo* under physiological loading remains to be understood.

Under the matching stiffness condition, the biodegradability of 7% GelMA with transglutaminase was comparable to 8% GelMA. This underlines an advantage of our strategy—that the addition of transglutaminase can stabilize the material in the defect *via* adhesion of GelMA to cartilage, rather than by increasing the concentration of the methacrylated hydrogel, and whilst maintaining the material's ability to be degraded by a biological enzyme (an intrinsic characteristic of collagen-based materials). While increasing the concentration of GelMA also saw higher push out strengths and thus increased stability of the gel in the cartilage ring, as discussed earlier, increasing the concentration of the material (and the subsequent impact on the time to degrade the material) can impair the formation of new tissue (36).

Future work in this area will focus on the impact transglutaminase has on stem cells undergoing chondrogenesis when encapsulated in the biomaterial. This is essential to understand not only the biocompatibility of the material, but also whether transglutaminase can support the formation of neocartilage and the integration of continuous matrix for long-term repair *in vivo*.

Fixation and subsequent integration is a challenge for many areas of soft tissue engineering and regenerative medicine, particularly those dealing with mechanical loads. This strategy has potential to be used in other tissues where bioadhesion may stabilize an implant, such as intervertebral disks, knee meniscus, or lung and cardiac tissues (51, 52). It may also aid the local delivery of drugs through adhesion to mucosa (53).

## Conclusion

Overall, this study provides a proof of concept for the addition of microbial transglutaminase to photocrosslinkable GelMA for adhesion to human cartilage. Transglutaminase enabled adhesion of the material to bovine and human cartilage tissue, and did not negatively affect the metabolic activity of hADSCs encapsulated in the material. The addition of transglutaminase also provides another mechanism for optimization of the mechanical and degradation properties of the scaffolds, likely due to the formation of a secondary network of covalent crosslinks. This bioadhesive material shows promise as a strategy to secure cell-laden scaffolds in cartilage defects, and warrants further investigation of its potential to support neocartilage generation and integration for long-term cartilage repair.

## Data Availability Statement

The raw data supporting the conclusions of this article will be made available upon request to the corresponding author, without undue reservation.

## Ethics Statement

The studies involving human participants were reviewed and approved by the Human Research Ethics Committee Research Governance Unit of St. Vincent's Hospital, Melbourne, Australia. The patients/participants provided their written informed consent to participate in this study.

## Author Contributions

AT designed the study, conducted the experiments, analyzed the data, and wrote the manuscript. SD and CO contributed to design of the study and execution of the *in vitro* cell experiment. SD, CO, and CO'C gave intellectual input throughout the project and contributed to analysis of the data. AO'C and CD contributed to conception and design of the study. All authors provided critical revisions of the manuscript and approved the final manuscript.

## Funding

This work was supported by The Victorian Medical Research Acceleration Fund (2018-Round 2), the Melbourne Medical School seeding grant, the Sylvia and Charles Viertel Charitable Foundation Clinical Investigator award, an Australian Government Research Training Program Scholarship, and an NHMRC-Medical Research Future Fund [App No. 1193897] Investigator Grant.

## Conflict of Interest

The authors declare that the research was conducted in the absence of any commercial or financial relationships that could be construed as a potential conflict of interest.

## Publisher's Note

All claims expressed in this article are solely those of the authors and do not necessarily represent those of their affiliated organizations, or those of the publisher, the editors and the reviewers. Any product that may be evaluated in this article, or claim that may be made by its manufacturer, is not guaranteed or endorsed by the publisher.
