# Supplementary material for: Microbial Transglutaminase Improves ex vivo Adhesion of Gelatin Methacryloyl Hydrogels to Human Cartilage
Source: Front Med Technol. 2021 Nov 18;3:773673. doi: 10.3389/fmedt.2021.773673 (PMC8757843; doi:10.3389/fmedt.2021.773673)
Supplement: Supplementary file 1 [file Data_Sheet_1.docx]

Supplementary Material

# Supplementary Figures

#
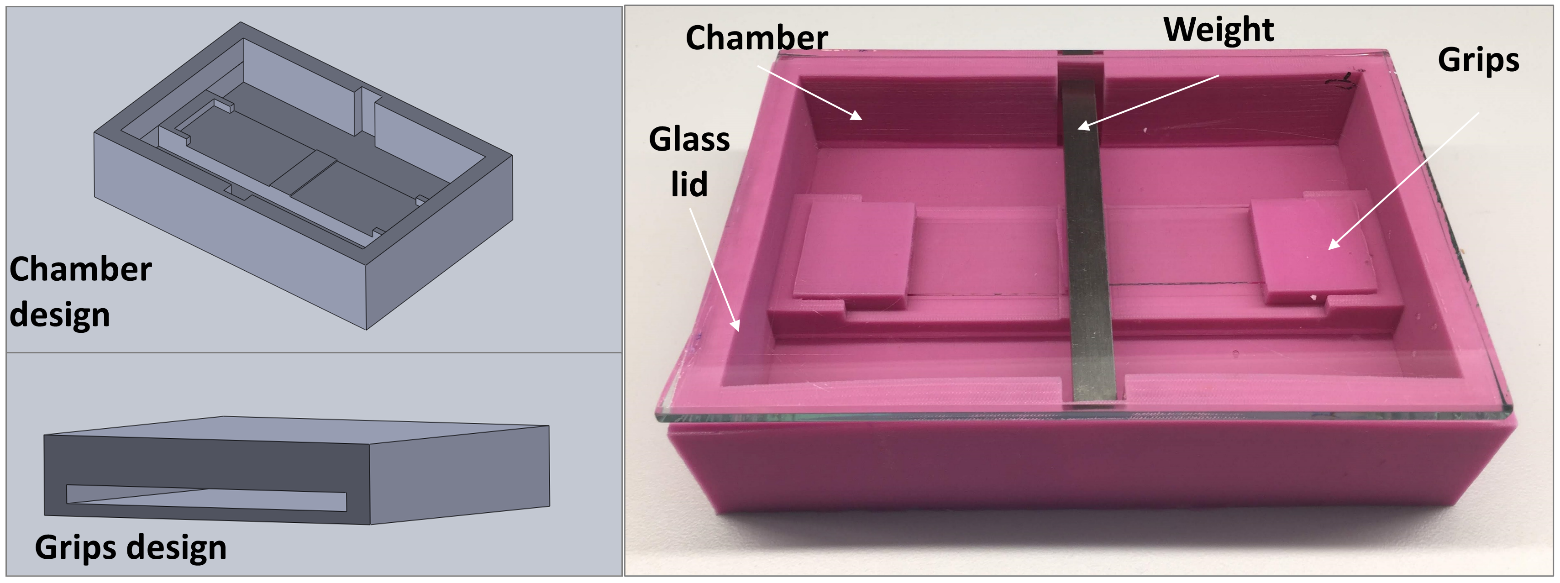


# Supplementary Figure 1. CAD designs of curing chamber and grips (left, top and bottom) for lap shear test, and silicone curing chamber with weight, glass lid and grips to offset sample supports.


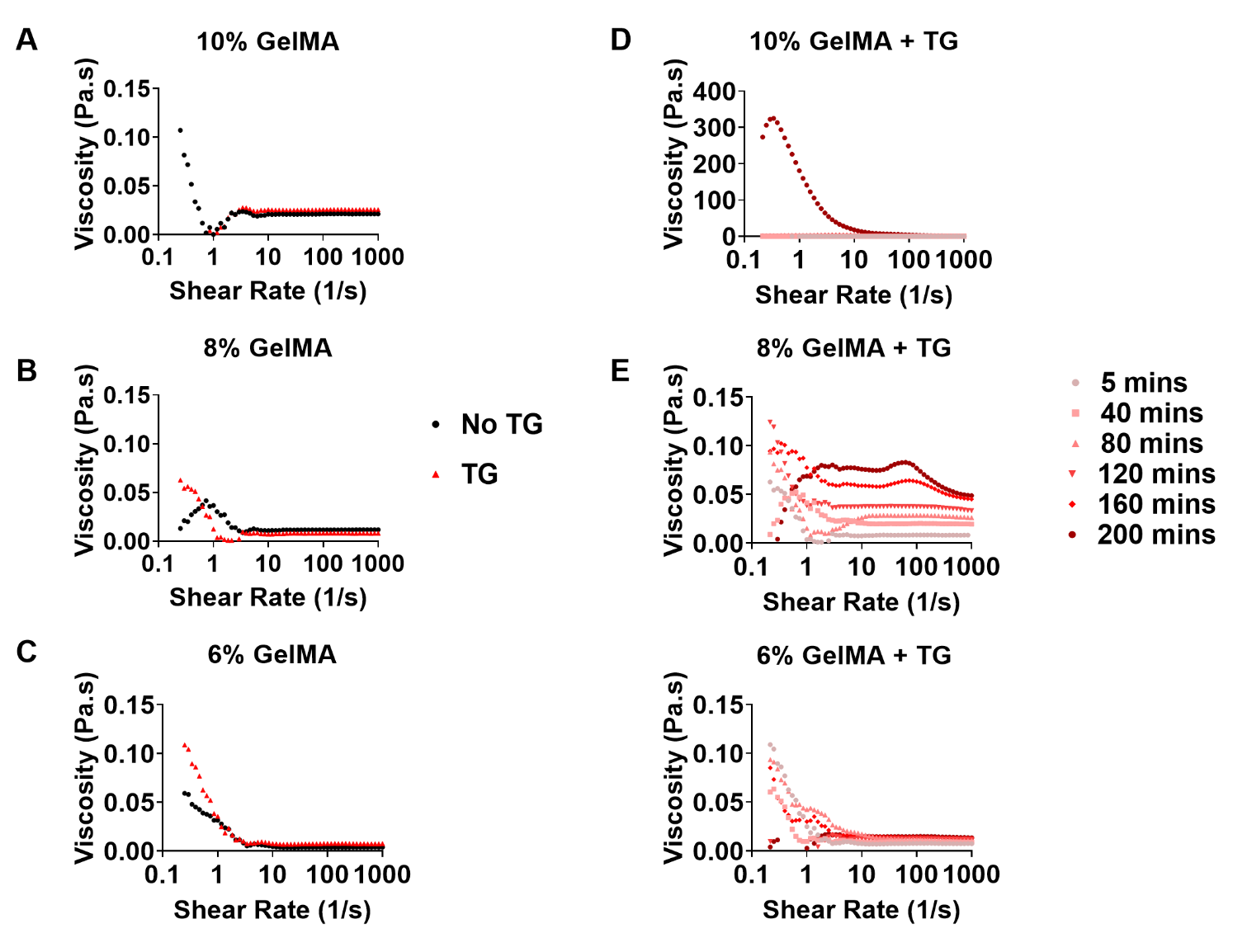


**Supplementary Figure 2.** Viscosity as a function of shear rate for three GelMA concentrations, with and without transglutaminase (A-C) and with transglutaminase after different intervals of incubation (D-F). After addition of transglutaminase and 5 minutes incubation at 37°C to ensure a homogeneous solution, all GelMA concentrations exhibited a similar viscosity over the range of shear rates tested to their corresponding controls without transglutaminase. A yield stress is observed for 10% GelMA after 200 minutes incubation. Though viscosity remains low for 8% and 6% GelMA, modulation of the viscosity is evident with increasing incubation time.


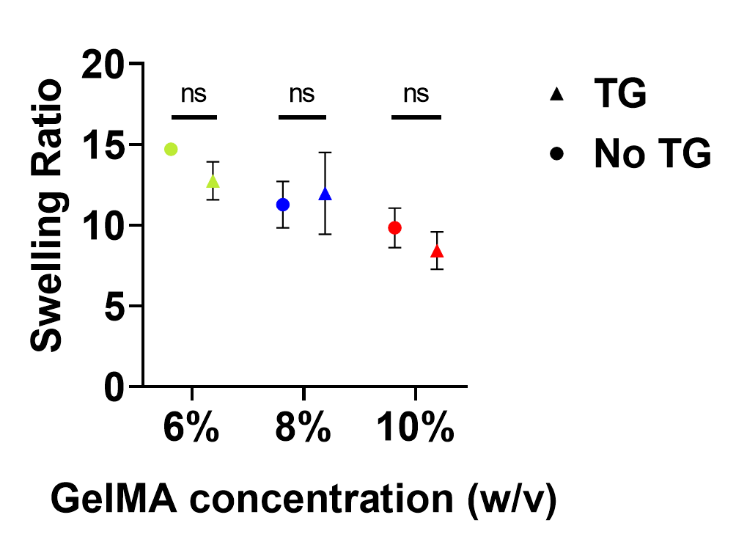


Supplementary Figure 3. Swelling ratio of GelMA hydrogels at three concentrations, with and without transglutaminase. Data points represent mean and standard deviation of three samples. The results show a trend of decreasing swelling ratio with increasing concentration of GelMA. Two-way ANOVA shows that the main effect of GelMA concentration is significant (p=0.0006), however the addition of transglutaminase did not have a significant main effect (p = 0.22). Post-hoc Bonferroni tests indicated no significant differences between the individual GelMA concentrations with and without transglutaminase.


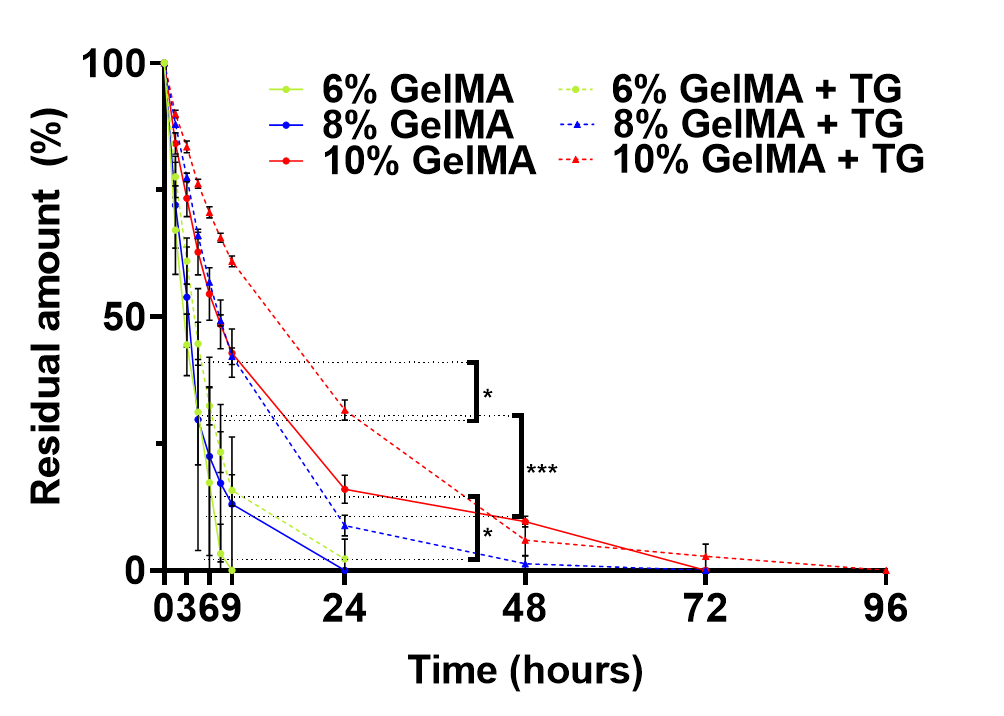


Supplementary Figure 4. Residual amounts of scaffolds remaining calculated via cumulative loss of fluorescent material for GelMA scaffolds at three concentrations, both without and with transglutaminase. Data points are means with standard deviation for three scaffolds for each condition.


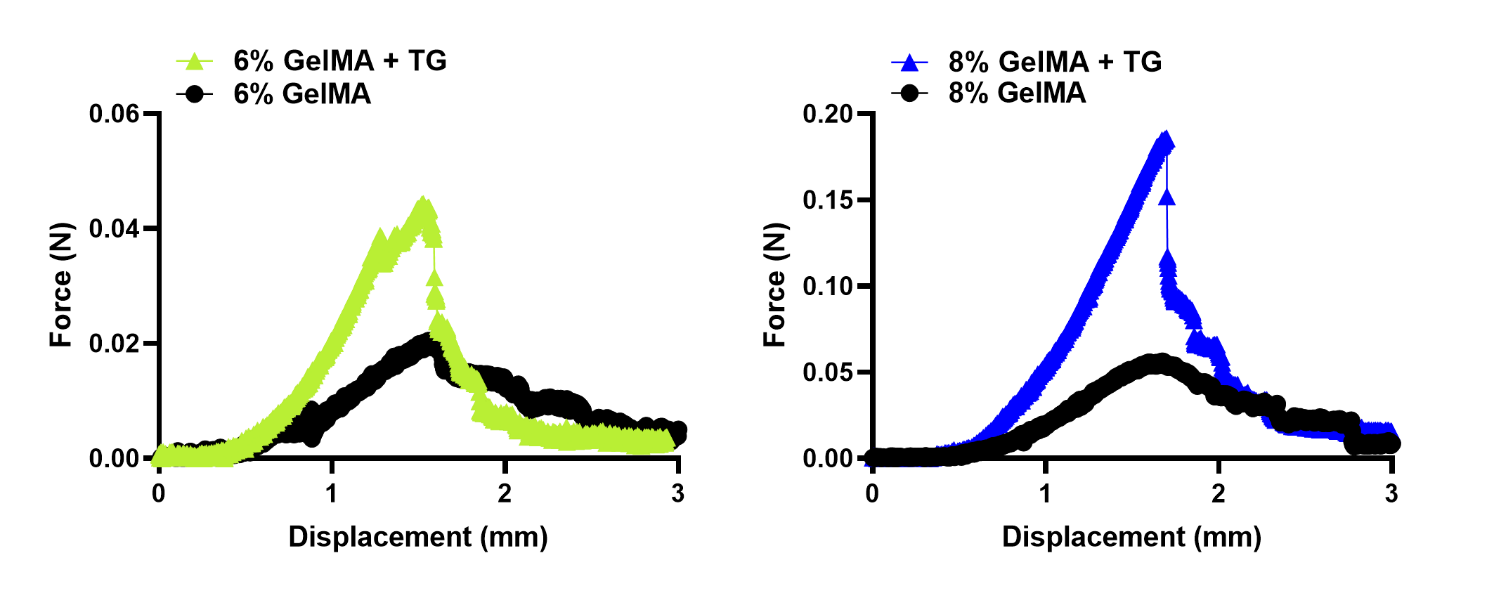


**Supplementary Figure 5.** Representative force-displacement curves for push-out tests for 6% (left) and 8% GelMA (right), with and without transglutaminase.


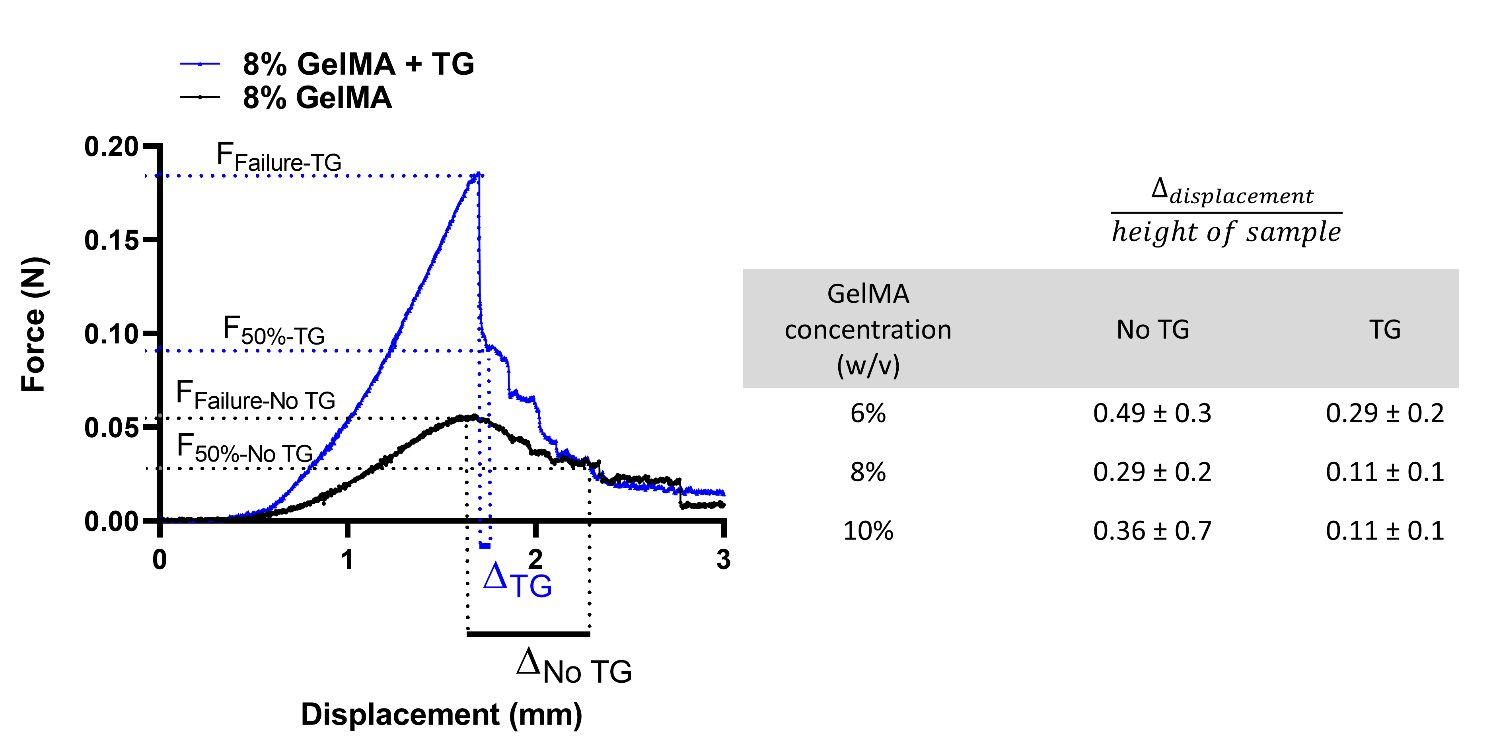


**Supplementary Figure 6.** Analysis of push-out test failure performed by calculating the strain from the peak failure force (F_failure_) to 50% of the peak failure force (F_50%_), as the change in displacement divided by the height of the scaffold in the cartilage ring. Table lists mean and standard deviation of the samples (at least 8 or more replicates). Samples containing transglutaminase saw a 50% drop in force over a smaller strain than those without, though variability is large. Two-way ANOVA finds that GelMA concentration does not have a significant effect on this measure, but that transglutaminase does have a significant main effect (p=0.0197). Post-hoc tests indicated no significant differences between the individual GelMA concentrations with and without transglutaminase.
